# What Combination of Generic Bedside Screening Tools Is Optimal to Capture Patients with Penetration/Aspiration Due to Dysphagia? Comparing Single Bedside Tools Versus Combinations of Tools for Sensitivity and Specificity

**DOI:** 10.3390/geriatrics10030063

**Published:** 2025-04-30

**Authors:** Albert Westergren, David Smithard, Johannes Riis, Christina Emborg, Anne Lund Krarup, Dorte Melgaard

**Affiliations:** 1 The PRO-CARE Group and the Research Platform for Collaboration for Health, Faculty of Health Sciences, Kristianstad University, 291 88 Kristianstad, Sweden; albert.westergren@hkr.se; 2 Elderly Care, Queen Elizabeth Hospital, Lewisham and Greenwich NHS Trust, London SE18 4QH, UK; david.smithard@nhs.net; 3 Centre for Exercise Activity and Rehabilitation (CEAR), School of Human Sciences, University of Greenwich, London SE9 2TB, UK; 4 Department of Geriatric Medicine, Aalborg University Hospital, 9000 Aalborg, Denmark; johannes.j@rn.dk; 5 Faculty of Clinical Medicine, Aalborg University, 9000 Aalborg, Denmark; apslk@rn.dk; 6 Nutritional Team, 4000 Roskilde, Denmark; christinaem@roskilde.dk; 7 EMRUn, Department of Acute Medicine and Trauma Care, Aalborg University Hospital, 9000 Aalborg, Denmark

**Keywords:** assessment, criterion related validity, dysphagia, eating difficulties, screening

## Abstract

**Background/Objectives**: This study aimed to explore the validity of various generic bedside screening tools, and combinations of these, for capturing dysphagia as compared to aspiration/penetration found through the Flexible Endoscopic Evaluation of Swallowing (FEES). **Methods**: In this cross-sectional study, participants diagnosed with chronic pulmonary disease (*n* = 25), Parkinson’s disease (*n* = 26), multiple sclerosis (*n* = 24), or stroke (*n* = 25) participated. Patient-reported outcomes and clinical-rated assessments included: the four-question test (4QT), the Minimal Eating Observation Form—II, the Volume–Viscosity Swallow Test (V-VST), the Penetration–Aspiration Scale, and the FEES. Activities in daily living were assessed with the Barthel Index. The sensitivity, specificity, negative predictive value (NPV), positive predictive value, and accuracy were calculated. **Results**: The 100 participants’ median age was 72 years, and 42 were women. In total, 78 patients had eating difficulties (MEOF-II). According to the 4QT, 69 patients had dysphagia while 62 had it according to the V-VST. Furthermore, 29 patients had penetration/aspiration according to the FEES. All generic bedside tools performed better when combined with another tool, when compared to the identification of penetration/aspiration according to the FEES. The combination of the MEOF-II and 4QT as well as the combination of the MEOF-II and V-VST proved to have very high sensitivity (96.1–96.3%) and NPVs (92.3% in both instances). Combining the three tools, the MEOF-II, 4QT, and V-VST, did not improve the sensitivity or NPV. **Conclusions**: A combination of the MEOF-II and 4QT or the MEOF-II and V-VST bedside tools is recommended for identifying patients at risk of penetration/aspiration and in need of further in-depth clinical assessment.

## 1. Introduction

Various screening tools have been developed to detect eating difficulties. Screening results are important since these can be used to select patients in need of further in-depth clinical assessment and at high risk of morbidity and mortality related to dysphagia. Clinical assessment might indicate a need for further assessment with invasive techniques, e.g., Videofluoroscopic Swallowing Study (VFSS)/Modified Barium Swallow (MBS), or with the Flexible Endoscopic Evaluation of Swallowing (FEES). Access to these kinds of invasive investigations can, in certain contexts, be limited or nonexistent. In addition, a recent study highlighted that it can be difficult to identify patients in need of further dysphagia assessment [1].

Tools used for dysphagia screening must have a high sensitivity to “rule-in” patients for whom in-depth clinical assessment and possibly invasive techniques, as described above, will be needed for identifying penetration/aspiration. When comparing screening tools against a gold standard, such as the FEES, “criterion-related validity” is relevant.. In a systematic review and meta-analysis, the Gugging Swallowing Screen (GUSS) provided sufficient data for a meta-analysis including three studies (*n* = 192 patients with acute stroke), demonstrating high sensitivity (96%) but low specificity (65%) as compared to the FEES [2]. In exploring the criterion-related validity, among the 134 participants, between the Volume–Viscosity Swallow Test (V-VST) and the Eating Assessment Tool (EAT-10), with the VFSS as the gold standard, sensitivity was found to be 94% and 85%, respectively [3]. In another study [4], among patients (*n* = 144) admitted to a public rehabilitation hospital, the sensitivity and specificity for the V-VST was 83.3% and 72.6%, respectively, and for the EAT-10, 82.8% and 57.5%, respectively, when using the FEES as the gold standard. It is reasonable for sensitivity to be given priority at the cost of specificity since over-identification is preferable to under-identification, given that positive screening results are followed by in-depth assessment. However, none of the studies [2,3,4] explored whether a combination of screening tools can improve the criterion validity. Thus, we hypothesized that a combination of bedside screening tools would yield better sensitivity than single tools compared to the FEES, serving as the gold standard. Therefore, the aim of this study was to measure the criterion-related validity of single generic bedside tools and of the following combination of bedside tools: (1) the Minimal Eating Observation Form—II (MEOF-II) and the four-question test (4QT); (2) the MEOF-II swallowing component (MEOF-IIs) and the 4QT; (3) the MEOF-II and the V-VST; (4) the MEOF-IIs and the V-VST; (5) the 4QT and the V-VST; (6) the MEOF-II, the 4QT, and the V-VST; and (7) the MEOF-IIs, the 4QT, and V-VST.

There are several reasons why this study is important. First, test costs (staff time) connected to screening for dysphagia must be considered. This is why it is important to find tools that are easy to use within a limited amount of time. Second, identifying which single tools or combinations of tools are best suited to identify patients with potential penetration/aspiration can indicate a need for in-depth assessment and potentially limit the number of patients in need of expensive (time and equipment), invasive assessments. Third, and most importantly, by identifying patients with dysphagia, and especially those at risk of penetration/aspiration, we can individually adapt rehabilitation and treatment. By doing so, we can possibly decrease morbidity and mortality and increase quality of life for patients.

Dysphagia is particularly prevalent among older adults and often underdiagnosed in geriatric care settings. Therefore, the evaluation of accessible and effective bedside screening tools has important implications for clinical practice in geriatrics.

## 2. Materials and Methods

This cross-sectional study, conducted at the North Denmark Regional Hospital in Hjørring, Denmark, adhered to the Declaration of Helsinki. The study received approval from the North Denmark Regional Ethics Committee on Health Research Ethics (approval number: N-20210026). All participants provided both oral and written informed consent before the study commenced. The study is reported following the COSMIN reporting guidelines [5]. The sample for this study has been used in a previous study [1] exploring the convergent and discriminant validity of the MEOF-II and different clinical characteristics.

### 2.1. Participants

Participants in this study were included and tested consecutively from 1 August 2021 to 31 September 2022. They were recruited from the community through newspaper advertisements and rehabilitation centers across various municipalities. Eligible participants included those diagnosed with chronic obstructive pulmonary disease (COPD), Parkinson’s disease (PD), or multiple sclerosis (MS) or those with sequelae from a stroke. These diagnostic groups were selected because they are frequently occurring and commonly experience eating difficulties and dysphagia [6,7,8,9]. Participants needed to be at least 18 years old, able to speak and understand Danish, and have sufficient energy levels to perform the tests. Exclusion criteria included cognitive impairment (inability to give informed consent) or severe dysphagia (requiring a feeding tube). Three individuals were excluded: two due to severe cognitive impairment and one for not meeting the diagnostic inclusion criteria. Recruitment continued until approximately 25 participants were included in each of the four diagnostic groups [1].

### 2.2. Data Collection

Demographic data (age, sex) and diagnoses were gathered through interviews. All participants completed standardized questionnaires, including the Barthel Index (BI) and the 4-question test (4QT). Each test was administered by the same trained assessor for all participants, though different assessors were assigned to each test. The participants underwent three validated clinical tests: the MEOF-II, the V-VST, and the FEES documented according to the Penetration–Aspiration Scale (PAS). A dysphagia therapist experienced in performing the FEES in intensive care and geriatric patients conducted the FEES and PAS scoring. Four different investigators administered the questionnaires and tests while blinded to the other results: one for the questionnaires, another for the MEOF-II, a third for the V-VST, and the last for the FEES. All clinical tests were conducted in a randomized order by trained occupational therapists experienced in dysphagia assessment, with the investigators blinded to the results. The MEOF-II and V-VST assessments were conducted separately by two occupational therapists specialized in dysphagia, ensuring consistency with a single examiner for each test [1].

### 2.3. Barthel Index (BI)

The BI is a tool used to assess functional ability across ten different areas, including personal hygiene and feeding, focusing primarily on activities. It is valuable for determining a patient’s need for assistance with daily living activities. Each of the ten items offers two to four response options, resulting in a total score ranging from 0 to 100, with higher scores indicating fewer difficulties with daily activities [1,10,11]. The total score was utilized in the analysis.

### 2.4. The Four-Question Test (4QT)

The 4QT is a four-question survey designed to screen older adults for dysphagia [1,12,13]. It primarily assesses the function of swallowing but also considers the activity of eating. The questions included in the survey are as follows:

1. Do you cough or choke when eating or drinking?
2. Does it take longer to finish your meals than it used to?
3. Have you altered the types of food you eat?
4. Does your voice change after eating or drinking?

Participants responded to each question with either “no problem” (scored as zero) or “having a problem” (scored as one). The total score ranges from zero to four, with higher scores indicating more-significant issues. In the analysis, having no problem was coded as 0 and having 1–4 problems as 1. The Danish version, 4QT-DK, has good validity and psychometric properties [12].

### 2.5. The Minimal Eating Observation Form—II (MEOF-II)

The MEOF-II is a screening instrument designed for the structured observation of eating difficulties across three categories: ingestion, deglutition/swallowing, and energy/appetite. It primarily assesses eating as an activity and swallowing as a body function. Each category includes three sub-questions, with a score of zero indicating normal eating and a score of one indicating difficulty. The MEOF-II allows for the calculation of category-specific scores (ranging from 0 to 3) and a total score (ranging from 0 to 9), with lower scores reflecting fewer problems [14]. The tool is psychometrically sound, demonstrating good validity and inter- and intra-rater reliability [14,15,16]. In the analysis, having no problem was coded as 0 and having 1–9 problems as 1. In addition, the MEOF-IIs was also used since it is more focused on swallowing, including the following three items: coping with food in the mouth; swallowing; and chewing. Having no problem was coded as 0 and having 1–3 problems as 1.

### 2.6. Volume–Viscosity Swallow Test (V-VST)

The V-VST is a bedside swallowing test used to assess the safety and effectiveness of swallowing various liquids (nectar, water, and pudding) in three volumes (5 mL, 10 mL, and 20 mL). This test specifically evaluates the body function of swallowing. The liquids were thickened using ThickenUp Clear, Nestlé Health Science (Vers-chez-les-Blancs, Switzerland), to achieve the desired consistencies. Indicators of decreased swallowing safety, such as changes in voice, a drop in oxygen saturation of 3% or more, and coughing, suggest an increased risk of aspiration. Impaired swallowing safety, indicated by coughing and reduced oxygen saturation, was recorded as dysphagia (no = 0; yes = 1) [1,17,18]. The V-VST is both a clinical tool for screening and a tool for the diagnosis of oropharyngeal dysphagia when instrumental assessment is not possible or available [19].

### 2.7. The Flexible Endoscopic Evaluation of Swallowing (FEES) and the Penetration–Aspiration Scale (PAS)

The FEES involves passing a laryngoscope through the nose to assess the anatomical structure of the pharynx and larynx, as well as the swallowing function. This procedure places specific focus on the body function of swallowing. An Olympus ENFP3 laryngoscope, connected to a CCD camera and color monitor, was utilized for the evaluation. Participants were given three different consistencies of liquid (nectar, water, and pudding) in three volumes (5 mL, 10 mL, and 20 mL). The same thickening agent as used in the V-VST, ThickenUp Clear, Nestlé Health Science (Vers-chez-les-Blancs, Switzerland), was employed to achieve the nectar and pudding consistencies. To enhance visibility, a blue food dye was added to the liquids. Patients were observed for coughing or any signs of penetration or aspiration. The results were recorded using the PAS (Penetration–Aspiration Scale), which ranges from one to eight, with specific verbal descriptions for each score (1 = material does not enter the airway, 8 = material enters the airway, passes below the vocal folds, no effort made to eject) [1,20,21]. In the analysis having no problem was coded as 0 and having a score of 2–8 as 1.

### 2.8. Analysis

The dichotomization of the screening instrument was carried out because this is often how the outcome is interpreted—that is, whether or not a problem is present. In the combined testing approach, a ‘yes’ indicating the presence of a problem in one or more of the tests was coded as a positive result. The diagnostic performance of the different screening tests was assessed by calculating the sensitivity, specificity, positive and negative predictive values (PPV and NPV, respectively), and accuracy [22,23], using results from the FEES as the gold standard. These indices provide values ranging from zero to one (or, equivalently expressed as a percentage), where higher values are preferred [22,23]. The screening tools in this study are designed to detect eating difficulties in general and dysphagia specifically. As such, it is reasonable to prioritize sensitivity and NPV over specificity, as over-identification is preferable to under-identification—especially considering that positive screening results are followed by in-depth assessments and further decisions regarding the need for invasive procedures [1,22,24]. Analyses were conducted using the free online tool MedCalc (https://www.medcalc.org/calc/diagnostic_test.php (accessed on 3 July 2024)).

### 2.9. Ethical Considerations

All participants provided written informed consent following the provision of both verbal and written information about the study. Ethical approval was granted by the Regional Ethics Committee of the Capital Region of North Denmark (*n*-20210026, 7 June 2021), and the study was registered with the Danish Data Protection Authority (F2023-116).

## 3. Results

### 3.1. Patient Characteristics

The sample characteristics are shown in Table 1 and included patients with stroke (*n* = 25), COPD (*n* = 25), MS (*n* = 24), and PD (*n* = 26). The median age of the participants (*n* = 100) was 72 years, and 42 were women. The median BI score was 90. Eating difficulties were found in 78 patients according to the MEOF-II. According to the MEOF-IIs, 38 patients had dysphagia, 69 patients had it according to the 4QT, and 62 had it according to the V-VST. Furthermore, 29 patients had penetration/aspiration according to the FEES, as documented according to the PAS (Table 1).

### 3.2. Criterion-Related Validity

**Figure 1:**
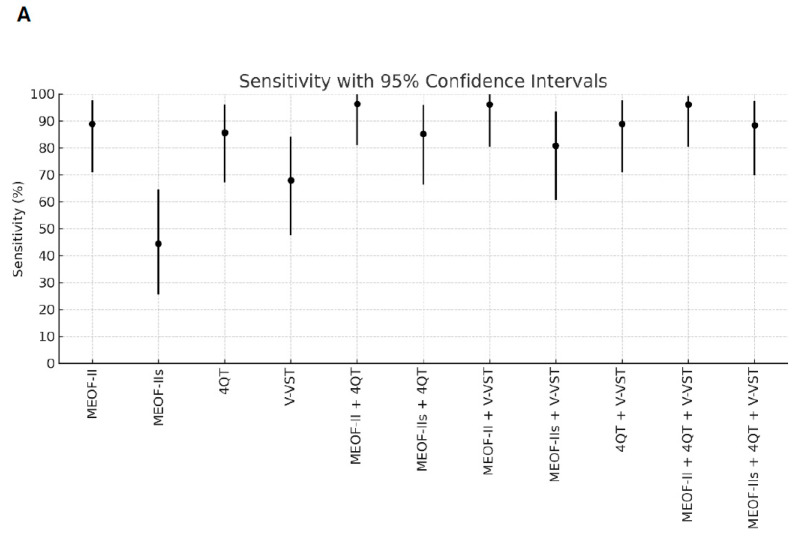

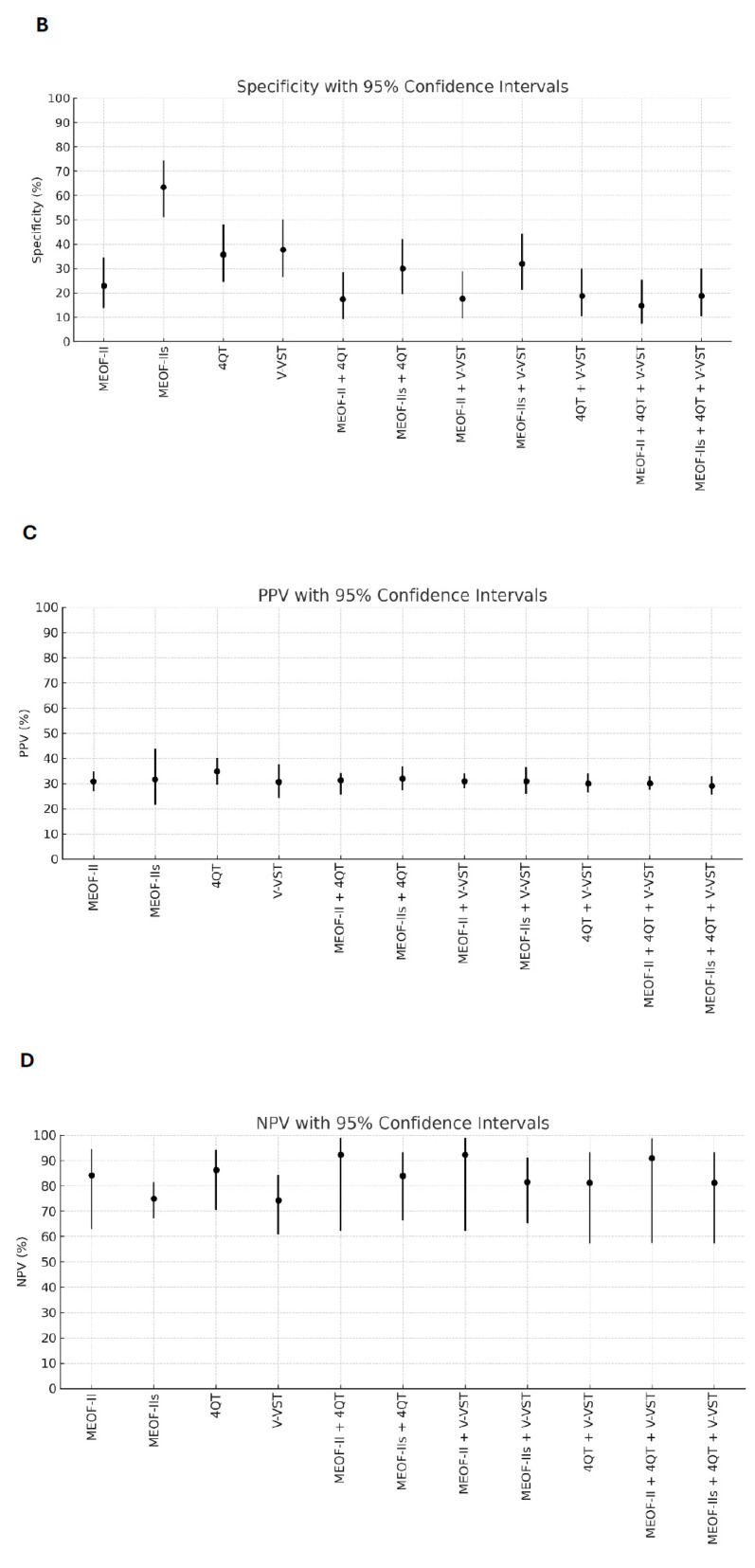

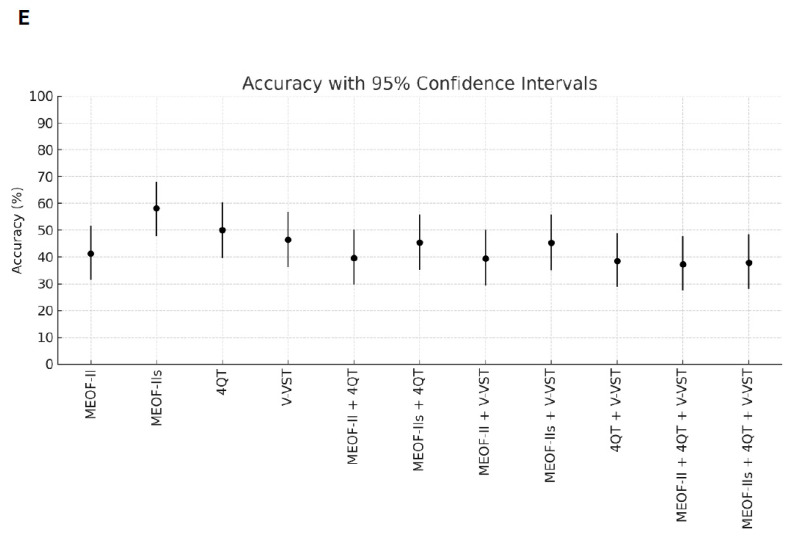
Sensitivity (Panel **A**), specificity (Panel **B**), positive predictive value (PPV) (Panel **C**), negative predictive value (NPV) (Panel **D**), and accuracy (Panel **E**) of single screening tests and combinations of tests with 95% confidence intervals. Abbreviations: MEOF-II = Minimal Eating Observation Form—II; MEOF-IIs = MEOF-II swallowing component; 4QT = a four-item questionnaire for screening older adults for dysphagia; V-VST = Volume–Viscosity Swallow Test.

Better sensitivity for detecting penetration/aspiration was found for all combinations of screening tools compared to single tools (Table 2). The best sensitivity (96.3%) for detecting penetration/aspiration was found in the combination of the MEOF-II and the 4QT. The best NPV was found in either the combination of the MEOF-II and the 4QT (92.3%) or the combination of the MEOF-II and the V-VST (92.3%). There was only one patient, corresponding to 1% of the sample, with penetration/aspiration that could not be identified by using any of the following combinations: the MEOF-II and the 4QT; the MEOF-II and the V-VST; or the MEOF-II, the 4QT and the V-VST. The best specificity and accuracy was found with the MEOF-IIs (63.4% and 58.2%, respectively), while the 4QT had the best PPV (34.8%) (Table 2 and Figure 1).

## 4. Discussion

To our knowledge, this is the first study of the criterion-related validity of combinations of generic bedside dysphagia screening tools, compared to the use of single tools, using the FEES as golden standard. Many patients (*n* = 78) were found to have eating difficulties in general and 29 patients were found to have penetration/aspiration according to the FEES. The results indicate that a combination of the MEOF-II and 4QT bedside tests seems to perform best for identifying patients in need of further assessment with the FEES.

Interestingly, we found only weak correlations between the MEOF-II, the V-VST, and the PAS in a previous study [1]. However, this was most likely due to the fact that the MEOF-II measures broader aspects of the ability to eat where the comparators, the V-VST and the PAS, focus on swallowing safety and effectiveness. In that study, it was hypothesized that different bedside tools used in combination would possibly increase the possibility of identifying candidates in need of the FEES.

In the clinic, there is a significant focus on the risk of aspiration associated with dysphagia, and with good reason, since it is associated with increased length of stay, morbidity, mortality, and decreased quality of life [25,26]. However, attention must also be given to reduced efficiency of eating, for instance, by using the MEOF-II. For example, this can cause patients to become fatigued before they are full, potentially leading to unplanned weight loss [27,28]. Similarly, certain types of food may be avoided because they are difficult to eat—often meat, for instance—resulting in an increased risk of inadequate protein intake [29]. This can lead to sarcopenia and reduced functional ability. Observing meals provides this type of insight, which is not achieved through instrumental examinations alone. In addition, generic bedside tests can be conducted anywhere, including nursing homes, private homes, and hospital wards. In contrast, instrumental examinations typically require specialized equipment that is usually only available in specialized departments. Thus, easy-to-use, validated, and reliable bedside tools need to be used in clinical practice to identify patients at risk of aspiration due to dysphagia.

Although this study included participants with varying diagnoses, the median age was 72 years, indicating that the findings are highly relevant to geriatric populations. Given the increased risk of dysphagia and its complications among older adults, implementing reliable bedside screening tools in geriatric care settings could significantly improve early detection and management. In addition, the current screening paradigm prioritizes the detection of aspiration risk, often at the expense of specificity. This approach, while suitable for identifying candidates for further instrumental assessment, may overlook functional abilities that are crucial for tailoring therapy. Including tools like the MEOF-II that focus on eating as an activity can help address this gap by identifying patients’ functional limitations and informing individualized care strategies.

The study showed that it is a good idea to combine subjectively reported problems (4QT) with objective observations of eating ability (MEOF-II), since this produces good sensitivity and negative predictive values. Furthermore, these are easy-to-use and cheap tests (in terms of staff time) that can be performed rather quickly by staff with very little training, thus requiring very few resources. The 4QT includes only four questions [12,13], and the time needed to conduct the nine-item MEOF-II screening is about 10 min or less when used as a part of the more extensive Minimal Eating Observation and Nutrition Form—Version II (MEONF-II) [30]. Either a combination of the MEOF-II and the V-VST or the 4QT and the MEOF-II is preferred. If patients can answer questions in a reliable way, the combination of the MEOF-II and the 4QT might be preferred. In cases where the patient has decreased ability to answer the questions in the 4QT, for instance, due to decreased cognitive ability or ability to communicate, one might instead use a combination of the MEOF-II and the V-VST.

In this study, lower specificity, for the screening tools as well as the assessment with the V-VST, was found compared to that in other studies [2,3,4]. For instance, the specificity in our study for the combination of the MEOF-II and the 4QT was 18 percent, and for the V-VST, it was 38 percent against penetration/aspiration according to the FEES. Interestingly, the MEOF-II demonstrated the highest specificity (63 percent) among both the individual screening tests and the combined test approaches. The lower specificity in our study might be due to different screening and assessment methods but also differences in sample characteristics. The sample in this study was rather heterogenous, while other studies had more homogenous samples. In one of the studies, with a specificity of 65 percent (Gugging Swallowing Screen vs. FEES), only patients with stroke were included for analysis [2]. In another study, with a specificity of 75 percent for impaired swallowing safety (V-VST vs. VFSS), only patients with a known history of dysphagia were included [3], while in a third study, patients with different kinds of motor physical sequelae were included, giving a specificity of 58 percent when comparing the Eating Assessment Tool 10 against the FEES, and 73% for the V-VST compared to the FEES [4]. The sample in our study might have had more chronic conditions causing more general eating difficulties [28], which, taken together, generated a higher number of false positives and thus produced lower specificity. Nonetheless, the low specificity remains a limitation, as it leads to a higher number of patients requiring in-depth assessments by staff trained in dysphagia evaluation—ultimately raising concerns about resource allocation.

It is a strength that the number of missing data was quite low (*n* = 0–3), with the exception of when several tests were combined; for instance, combining the MEOF-II and the 4QT produced missing responses for four patients, and combining both the MEOF-II and the V-VST and the MEOF-II, the 4QT, and the V-VST produced missing data for six patients. It is also a strength that we included patients with different conditions, increasing the generalizability of our results across patient groups. But it is a limitation that the sizes of the subgroups with different diagnoses does not allow for subgroup analysis [1]. In addition, the overall design was strong, with tests performed by different, clinically experienced raters blinded to each other’s results. It is also a strength that bedside tests and self-reports were tested in combination with instrumental observations (FEES). Although each test was performed by one trained assessor to ensure consistency, we acknowledge that using a single rater, especially for the FEES, may limit the external validity and obscure potential variation in clinical ratings across assessors.

The dichotomization of test outcomes reflected the primary aim of the study: to determine whether a positive screen (presence of any problem) was predictive of penetration/aspiration as assessed by the FEES. This approach aligns with clinical practice, where binary screening results guide further diagnostic workup. We acknowledge that this procedure simplifies complex phenomena and that the thresholds for ‘problem’ versus ‘no problem’ may be viewed as somewhat arbitrary. Future studies may build on this work by applying more-nuanced analyses using ordinal scores or continuous metrics to explore gradations of impairment and potentially more-precise predictive patterns.

## 5. Conclusions

In conclusion, combining generic bedside tools—either the MEOF-II with the 4QT or the MEOF-II with the V-VST—is recommended for identifying patients at risk of penetration or aspiration and who may require further in-depth assessment, potentially followed by instrumental evaluations such as the FEES. The MEOF-II and 4QT combination can be used for patients who are able to communicate and are cognitively intact. In other cases, a combination of the MEOF-II and the V-VST might be preferable. These findings are especially relevant for geriatric care, where resource constraints often limit access to instrumental swallowing assessments, and where early identification of dysphagia can greatly impact health outcomes in older adults.

## Figures and Tables

**Table 1:** Patient characteristics.

	All Patients, *n* = 100
Age, median (q1–q3)	72 (63–77)
Female/male, *n*	42/58
Activities of daily living, Barthel Index, high score = better, median (q1–q3)	90 (60–100)
MEOF–II, low score = better	
Food intake, median (q1–q3)	0 (0–1)
Food intake problems, *n*	32
Swallowing, median (q1–q3)	0 (0–2)
Swallowing problems, *n*	38
Energy/appetite, median (q1–q3)	1 (0–1)
Energy/appetite problems, *n*	64
Total score, median (q1–q3)	2 (1–4)
Challenged in one or more eating categories (intake/swallowing/energy), *n*	
One category	35
Two categories	30
Three categories	13
Any problems, *n*	78
Dysphagia, 4QT, low score = better, median (q1–q3)	2 (0–3)
Any problems, *n*	69
Volume–Viscosity Swallow Test, V-VST	
Dysphagia, *n*	62
Viscosity, managing unmodified/nectar/pudding, *n*	89/7/1
Volume, managing high (20 mL)/middle (10 mL)/low (5 mL), *n*	63/26/8
Penetration–Aspiration, PAS, low score = better, median (q1–q3)	1 (1–1.2)
Having penetration/aspiration, *n*	29

Missing data varies between 0 and 3.

**Table 2:** Sensitivity, specificity, positive predictive value (PPV), negative predictive value (NPV), and accuracy from single screening tests and combinations of tests. **Bold** figures highlight the highest values within each column.

		Aspiration/Penetration Assessed Through the Flexible Endoscopic Evaluation of Swallowing (FEES) and Documented According to the Penetration–Aspiration Scale (PAS)						
Screening Tests	Problem Bedside	Yes, *n* = 27	No, *n* = 70	Sens (95% CI)	Spec (95% CI)	PPV (95% CI)	NPV (95% CI)	Accuracy (95% CI)
MEOF-II	Yes	24	54	88.9 (70.8–97.6)	22.9 (13.7–34.4)	30.8 (27.0–34.8)	84.2 (62.8–94.4)	41.2 (31.3–51.7)
	No	3	16					
MEOF-IIs	Yes	12	26	44.4 (25.5–64.7)	**63.4 (51.1–74.5)**	31.6 (21.5–43.7)	75.0 (67.2–81.4)	**58.2 (47.8–68.0)**
	No	15	45					
4QT	Yes	24	45	85.7 (67.3–96.0)	35.7 (24.6–48.1)	**34.8 (29.7–40.2)**	86.2 (70.5–94.2)	50.0 (39.7–60.3)
	No	4	25					
V-VST (dysphagia)	Yes	19	43	67.9 (47.6–84.1)	37.7 (26.3–50.2)	30.6 (24.4–37.7)	74.3 (60.9–84.3)	46.4 (36.2–56.8)
	No	9	26					
**Combined tests**								
MEOF-II and 4QT	Yes	26	57	**96.3 (81.0–99.9)**	17.4 (9.3–28.4)	31.3 (25.6–34.2)	**92.3 (62.1–98.9)**	39.6 (29.7–50.1)
	No	1	12					
MEOF-IIs and 4QT	Yes	23	49	85.2 (66.3–95.8)	30.0 (19.6–42.1)	31.9 (27.4–36.9)	84.0 (66.5–93.3)	45.4 (35.2–55.8)
	No	4	21					
MEOF-II and V-VST	Yes	25	56	96.1 (80.4–99.9)	17.6 (9.5–28.8)	30.9 (28.1–33.8)	**92.3 (62.1–98.9)**	39.4 (29.4–50.0)
	No	1	12					
MEOF-IIs and V-VST	Yes	21	47	80.8 (60.6–93.4)	31.9 (21.2–44.2)	30.9 (25.9–36.4)	81.5 (65.1–91.2)	45.3 (35.0–55.8)
	No	5	22					
4QT and V-VST	Yes	24	56	88.9 (70.8–97.6)	18.8 (10.4–30.1)	30.0 (26.4–33.8)	81.2 (57.3–93.3)	38.5 (28.8–49.0)
	No	3	13					
MEOF-II, 4QT, and V-VST	Yes	25	58	96.1 (80.4–99.3)	14.7 (7.3–25.4)	30.1 (27.5–32.8)	90.9 (57.4–98.7)	37.2 (27.5–47.8)
	No	1	10					
MEOF-IIs, 4QT, and V-VST	Yes	23	56	88.4 (69.8–97.5)	18.8 (10.4–30.1)	29.1 (25.5–32.9)	81.2 (57.3–93.3)	37.9 (28.1–48.4)
	No	3	13					

MEOF-II = The Minimal Eating Observation Form – II; MEOF-IIs = MEOF-II swallowing component; 4QT = a four-item questionnaire for screening the older adults for dysphagia; V-VST = Volume–Viscosity Swallow Test (V-VST). Missing data varies between 2 and 6.

## Data Availability

Data are unavailable due to GPDR restrictions.

## Abbreviations

The following abbreviations are used in this manuscript:

BI	Barthel Index
FEES	Flexible Endoscopic Evaluation of Swallowing
MEOF-II	Minimal Eating Observation Form—Version II
MEOF-IIs	Minimal Eating Observation Form—Version II, swallowing component
MS	Multiple Sclerosis
PAS	Penetration–Aspiration Scale
PD	Parkinson’s disease
V-VST	Volume–Viscosity Swallow Test
4QT	Four-question test
